# Genetic Evaluation and Screening in Cardiomyopathies: Opportunities and Challenges for Personalized Medicine

**DOI:** 10.3390/jpm13060887

**Published:** 2023-05-24

**Authors:** Sahana Aiyer, Emilia Kalutskaya, Arianne C. Agdamag, W. H. Wilson Tang

**Affiliations:** 1 Department of Cardiovascular and Metabolic Sciences, Lerner Research Institute, Cleveland Clinic, Cleveland, OH 44195, USA; 2 Boonshoft School of Medicine, Wright State University, Fairborn, OH 45435, USA; 3 Department of Cardiovascular Medicine, Heart Vascular and Thoracic Institute, Cleveland Clinic, Cleveland, OH 44195, USA

**Keywords:** genetic testing, cascade testing, cardiomyopathy

## Abstract

Cardiomyopathy is a major cause of heart failure caused by abnormalities of the heart muscles that make it harder for it to fill or eject blood. With technological advances, it is important for patients and families to understand that there are potential monogenic etiologies of cardiomyopathy. A multidisciplinary approach to clinical genetic screening for cardiomyopathies involving genetic counseling and clinical genetic testing is beneficial for patients and families. With early identification of inherited cardiomyopathy, patients can initiate guideline-directed medical therapies earlier, resulting in a greater likelihood of improving prognoses and health outcomes. Identifying impactful genetic variants will also allow for cascade testing to determine at-risk family members through clinical (phenotype) screening and risk stratification. Addressing genetic variants of uncertain significance and causative variants that may change in pathogenicity is also important to consider. This review will dive into the clinical genetic testing approaches for the various cardiomyopathies, the significance of early detection and treatment, the value of family screening, the personalized treatment process associated with genetic evaluation, and current strategies for clinical genetic testing outreach.

## 1. Introduction

Cardiovascular disease (CVD) remains the leading cause of death, accounting for 71% of deaths globally each year [1]. Inherited CVD, caused by genes passed down through families, includes but is not limited to cardiomyopathies, arrhythmias, cardiac tumors, heart valve diseases, pulmonary hypertension, and so forth [2]. Recent evidence has uncovered new inherited cardiovascular disease genes and their contributions in disease progression. It is imperative to investigate inherited cardiovascular disease and strategies to help diagnose such diseases at an early age to initiate treatment for individuals. Since preventative medicine is an integral part of health care, implementing strategies to educate, outreach, and encourage healthy behaviors in various communities helps patients prevent long-term health issues.

Genetic testing for cardiomyopathies plays an increasingly important role in the clinical care of patients and has been increasingly adopted in contemporary cardiology practice. Genetic variants have been implicated in 25–40% of patients with dilated cardiomyopathy with a positive family history, but also in 10–30% of patients without a recognized family history [3,4]. Phenotype and family history are important for identifying patients in whom clinical genetic testing is most likely to yield clinically actionable information. Presentation of cardiomyopathy with conduction disease or ventricular arrhythmias, especially with early disease onset, often prompts suspicion of a genetic etiology. According to the latest clinical practice guidelines in heart failure for selected patients with cardiomyopathy (especially with no known ischemic etiology), referral for genetic counseling and testing is reasonable to identify conditions that could guide treatment for patients and family members (Class IIa, Level of evidence B) [5]. In first-degree relatives of selected patients with genetic or inherited cardiomyopathies, genetic screening and counseling are recommended to detect cardiac disease and prompt consideration of treatments to decrease heart failure progression and sudden death (Class I, Level of evidence B) [5]. The results of clinical genetic testing have allowed for the identification of pathogenic variants linked to inherited cardiomyopathies [6]. With genetic evaluation and testing, the specific pathogenic variants can be targeted with better appreciation of the clinical trajectories, thereby helping to streamline diagnostic testing, screening, genetic counseling, and personalized care processes [6]. It is important to incorporate genetic evaluations into a multidisciplinary care approach and continue to advance novel strategies for clinical genetic testing of cardiomyopathies.

## 2. Testing Strategies

There is increasing recognition that family history is often incomplete and that de novo genetic variants may occur. Clinicians are unable to identify all patients with genetic cardiomyopathy solely through a three-generation family history. For example, Dellefave-Castillo and colleagues found that if only patients highly suspected of genetic cardiomyopathy or arrhythmia were genetically tested, over 137 positive results (14.4%) would have been missed [7]. Similarly, 10.9% of positive results would have been missed if genetic testing was only performed on panels with any diagnostic indications [7]. Therefore, clinical genetic evaluation should be considered in every patient presenting with unexplained cardiomyopathies (and perhaps soon on all cardiomyopathies). The steps involved in clinical genetic evaluation for cardiomyopathy include: (1) documenting a thorough and detailed family history; (2) individualized patient counseling to address specific concerns; and (3) molecular genetic testing, commonly through established test panels of known cardiac monogenic variants [8]. Discussion of family history involves taking a three-generation family history, examining potential inheritance and penetrance patterns, conveying implications of genetic results on medical treatment, and considering the potential of cascade testing for relatives [6].

Molecular genetic testing consists of disease-specific panels, targeted variant tests, broader cardiac panels, whole-exome sequencing (WES), and whole-genome sequencing (WGS) [6]. Disease-specific panels involve genes that have a high or moderately high link to the disease of interest [6]. Examples include hypertrophic cardiomyopathy (HCM)- or dilated cardiomyopathy (DCM)-specific panels. Broader cardiac panels may involve genes with a lower prevalence or lower correlation to phenotypes of interest [6]. WES genetically sequences the entire DNA’s protein-coding regions (exons), while WGS sequences not only exons, but also the DNA’s noncoding regions (introns) and mitochondrial DNA [6]. The cardiac gene testing panels and their pathogenic/likely pathogenic genetic variants that clinicians use in clinical practice are focused on Mendelian monogenic diseases in which there has been established phenotype–genotype associations.

A comparison of disease-specific gene panels and broader gene panels may provide some insights into their clinical applications. Broader gene panels are likely to increase the detection rates of genes, but can also identify variants of uncertain significance (VUSs) [6]. Ouellette and colleagues found that broad cardiomyopathy panel testing had a significantly higher rate of detecting VUS compared with a targeted disease-specific panel (87% vs. 30%) [9]. The broad cardiomyopathy panel test only identified pathogenic variants in genes that overlapped with the targeted panels [9]. Detecting individual VUSs may stir ambiguity and confusion for health professionals and families of patients, which are potentially undesired consequences of broader gene testing [10].

Many WESs and WGSs are still utilized for research purposes over clinical use when evaluating for inherited cardiomyopathies [6]. A study shows that WES could detect likely pathogenic or pathogenic variants for almost half of HCM patients [11]. WES can also detect secondary findings unrelated to the primary indication for testing, such as cancer susceptibility [11]. In Ouellette’s study, WES identified 84 secondary findings for HCM patients [11]. WGS is instrumental in detecting deep intronic gene variants; recent evidence has demonstrated intronic variants to be pathogenic [12]. A study found that with WGS, deep intronic splice variants in MYBPC3 were found in 4 out of 46 individuals with previously negative HCM genetic test results [12]. With the broader coverage and improved diagnostic capacity of WES and WGS, these genetic sequencing methods should be implemented in large families with many disease-affected individuals and relatives [6].

Interpretation of genetic variants is based on published guidelines by the American College of Medical Genetics (ACMG) [13]. The categories of genetic variants are: benign, likely benign, VUS, likely pathogenic, and pathogenic (Table 1) [6]. The literature and data are constantly evolving, so variants are likely to be reinterpreted over time, with VUS changing to likely pathogenic/pathogenic and pathogenic variants changing to VUS [6]. Of the genes determined to be “clinically actionable” by the ACMG, half are related to cardiovascular diseases. However, due in part to high levels of uncertainty surrounding cardiovascular genetics, there is still some disagreement within the field about how to order and interpret these tests. However, clinically available genetic testing has matured over the past decade to focus on a subset of ~40–100 cardiac genes with relatively well-established genetic variants that are linked to clinical cardiomyopathy and arrhythmia manifestations. This has been rigorously curated through careful adjudication of the clinical significance of variants that are submitted by clinical testing laboratories, research laboratories, expert panels, and other groups and shared publicly in ClinVar. ClinVar, a public database of variants and their connection to phenotype and disease, is commonly used to classify variants [6]. Interpretation of genetic variants needs to be thorough to integrate the most recent data and evidence [6]. It is common for VUSs to be detected in patients with no significant family history [6]. With new data available over time, it is advised that the medical care team contact the patient again in a few years to evaluate any updates regarding the VUS to prevent missing any pathogenic variants [6]. Clinicians should also be available to help counsel patients when genetic variants are being reclassified as pathogenic or likely pathogenic. New technologies are developing to share recent updates and information on variant classification with families such as the CardioClassifier tool and a genotype update system [14,15].

## 3. Cascade Family Testing

**Figure 1:**
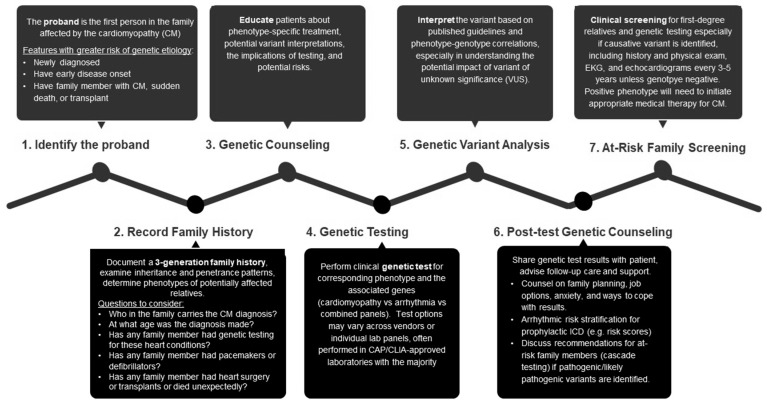
Genetic Evaluation of Specific Cardiomyopathies and Cascade Testing Strategy.

Proband (in this case the patient with cardiomyopathy) clinical genetic testing occurs when the first person in a family is affected by a genetic condition and receives genetic counseling (Figure 1) [6]. Once a pathogenic gene is identified, information regarding a potential genetic variant that may affect people across generations of family members should be relayed to those at risk [6]. Genetic counselors and specialists collaborate with patients to construct a communication plan to contact relatives and inform them about the genetic condition and its implications, including performing genetic and cardiac screening [6]. If the proband patient expresses a VUS or any negative result, relatives are still urged to participate in continuous clinical screening since they may express the phenotype [6].

Reclassification of genetic variants plays a major role in screening guidelines for cascade testing [6]. When a likely pathogenic or true pathogenic variant is reclassified as VUS, relatives who previously tested negative for the variant would have been removed from close monitoring [6]. These relatives must still be counseled appropriately and re-contacted [6]. Likewise, VUSs altering to likely pathogenic/pathogenic variants need to be accounted for so that relatives at risk can participate in cascade testing and screening [6]. These genetic variant reclassifications can cause confusion for patients and their families. Appropriate genetic counseling by a multidisciplinary team is critical to address these potential issues [16].

## 4. Clinical Genetic Testing Impact on Treatment

Recent advances in clinical genetic testing have improved understanding in this field, allowing for improvement in the care of cardiomyopathy patients and their families. The association between genotypes and phenotypes for cardiomyopathies may vary widely, but recent evidence is evolving scientific understanding. There is a level of genetic heterogeneity, as variants in specific genes can result in different phenotypes [6]. For example, variants in the nuclear envelope protein lamin A (LMNA) gene can lead to progressive DCM or arrhythmogenic cardiomyopathy (ACM), while variants in the MYH7 gene can lead to DCM, HCM, restrictive cardiomyopathy (RCM), or left ventricular non-compaction cardiomyopathy (LVNC) [17,18]. However, many gene variants are specific to a singular phenotype, making the association between genotypes and phenotypes more challenging [6].

Genetic testing is useful for diagnosing and improving treatment strategies [6]. Among HCM patients, genetic testing allows for the differentiation of primary cardiomyopathies from phenocopies such as transthyretin (TTR)-cardiac amyloidosis, Fabry disease, and glycogen storage diseases [19]. By detecting high-risk individuals earlier, targeted therapies can be initiated sooner to prevent the progression of cardiomyopathies, including early reverse remodeling therapies and primary prophylaxis implantable cardiac defibrillators (ICDs), in high-risk patients [5]. For DCM patients, starting guideline-directed medical therapy with beta-blockers, mineralocorticoid receptor antagonists, angiotensin receptor neprilysin inhibitors, and sodium-glucose cotransporter 2 inhibitors can aid in preventing disease progression, while using calcium-channel blockers and disopyramide for HCM can help improve symptoms [20,21]. These highlight the role of clinical genetic testing in the early implementation of personalized disease-specific treatment.

## 5. Emerging Strategies

Recent efforts to expand WES and WGS have shown the potential of providing incidental findings of actionable results related to cardiac-related gene variants when testing a patient with a negative cardiac family history [6]. The ACMG recommends that patients who have undergone WES and WGS consider investigating if they express likely pathogenic/pathogenic variant genes associated with cardiovascular disease that are clinically actionable [22]. In a study assessing 2628 elderly individuals subjected to WES, 11 were found to express pathogenic variant genes linked to cardiomyopathies [23]. Only 2 of these 11 individuals demonstrated evidence of the associated disease after analyzing 25 years of medical chart coding data during follow-up [23]. These results indicate that among genotype-positive patients, only a small percentage demonstrate overt cardiovascular phenotypes [20].

Polygenic risk scores (PRSs) are currently being utilized to better understand disease penetrance of monogenic variants [24]. PRSs consist of thousands of single nucleotide polymorphisms (SNPs) that signify risk variants which help detect high-risk individuals [24]. PRSs are useful in interpreting cardiac genetic testing to help at-risk family members [24]. Although some PRSs have been developed to identify those at risk of developing heart failure or therapeutic responses, routine clinical use is lacking since the majority of the cardiovascular PRSs have not been approved for clinical use.

With the increasing number of VUSs, researchers are expanding functional assays to help clarify the pathogenicity of variants [25,26]. These functional assays have the potential to analyze cell size, contractility, action potentials, sarcomere structure, and gene expression [26]. Although functional assays show promising potential, they cannot be used on a large scale due to their high cost [26]. Other potential technologies to assist in gene and drug therapies include CRISPR-Cas9, gene replacement, allele-specific silencing, and exon skipping [26]. While these genome editing techniques are not available for clinical use, they signify great potential by targeting the genetic causes of disease. These precision medicine strategies demonstrate exciting potential in the treatment of genetic cardiomyopathies.

## 6. Genetic Evaluation and Clinical Management of Specific Cardiomyopathies

The presence of likely pathogenic or pathogenic variants, while continuing to evolve, has been broadly adopted by clinicians and clinical genetic specialists for identifying those with suspected inherited cardiomyopathy in which appropriate diagnostic testing and interventions are sought, especially in those that may have a known malignant natural history. On the other hand, there are clinicians that believe increased genetic data can lead to confusion and inappropriate treatment due to the uncertainty of the results and their treatment implications. In addition, the poor diversity of genetic reference databases still critically limits our ability to interpret genetic findings in underrepresented populations. It is true that we still do not have an in-depth understanding of all the genetic predispositions for various cardiomyopathies and continue to discover new aspects of genetic underpinnings of cardiovascular diseases. In this section, we highlight some emerging data that illustrate the opportunities and challenges of personalized medicine in inherited cardiomyopathy.

### 6.1. Dilated and Arrhythmogenic Cardiomyopathy

DCM can be categorized as either familial or nonfamilial [8]. More than 50 genes have been correlated to DCM; 23 of these genes are accountable for almost all types of genetic DCM [3]. These DCM genes regulate various cardiomyocyte structures such as the desmosome, cytoskeleton, sarcomere, nuclear lamina, and mitochondria [8]. Among patients with DCM, those with positive genotypes tend to exhibit increased heart failure, arrhythmia, and worse left ventricular reverse remodeling compared with those with negative genotypes [27]. Detecting a specific variant will not greatly change the course of treatment for all DCM patients, but there are some important exceptions [8]. It is imperative to be aware of certain DCM genotypes that require unique medical management and treatment [8].

The most common genetic causes of DCM are the titin (TTN) gene variants [28]. Truncating variants of the TTN gene (TTNtv) are associated with 25% of familial DCM cases and 18% of sporadic DCM cases [28]. With TTN-associated DCM, deterioration is quicker than with non-TTN DCM [28]. Even though many patients with TTN have good responses to drug therapies, those with TTNtv may experience earlier death and a higher risk of transplantation and ventricular assist devices compared with those with non-TTNtv [28]. It is important to recognize that TTN was not evaluated in patients who underwent genetic testing before 2012 because it was not included in first-generation genetic panels; hence, updated genetic test panels should be repeated in those with earlier genetic evaluations [28]. Although truncated variants are indicative of genetic DCM, they are also found in about 1–3% of the normal population, especially in locations closer to the Z-disk region [28]. Future research should focus on recognizing unique factors related to pathogenicity in TTNtv.

LMNA is another important gene strongly associated with DCM [29]. In addition to heart failure, LMNA causes debilitating bradyarrhythmia (high grade heart blocks) and tachyarrhythmia (atrial fibrillation or ventricular tachycardia) [29]. Therapies are focused on preventing sudden death and heart failure progression as well as implementing primary prevention ICDs in those with causative LMNA variants, even though many may be refractory to medical therapy [29]. Some patients who partake in competitive sports and rigorous activity may exhibit worse health outcomes; hence, these patients are specifically advised on personalized exercise modifications [29]. Carriers of LMNA variants should also consult with neuromuscular specialists, as certain variants are associated with skeletal myopathy and creatine kinase elevation [29]. Currently, there are no targeted drug therapies for LMNA-associated DCM [29], and recent clinical trials investigating a small molecule inhibitor of an MAPK pathway in treating LMNA-associated DCM have failed to improve functional capacity [30]. 

SCN5A is another gene linked to DCM and is associated with various deleterious cardiac phenotypes such as familial atrial fibrillation, Brugada syndrome, and familial conduction disease [31]. SCN5A is a specific genotype that may require unique medical management [31]. Treatment strategies for other cardiomyopathies may not hold the same efficacy for SCNA5-associated DCM [31]. For example, sodium-channel blockers are a successful precision medicine strategy, but they counteract in SCNA5-associated DCM by exerting proarrhythmic effects [32].

Approximately 10% of those with genetic DCM have damaged variants in genes encoding for desmosomes [27]. These genes are generally linked to arrhythmogenic right ventricular cardiomyopathy (ARVC), but recent evidence has correlated some of them (such as desmoplakin [DSP]) to biventricular cardiomyopathy as well [33]. Desmosomal genes are correlated with higher rates of malignant ventricular arrhythmia, but decreased left ventricular reverse remodeling compared with other DCM genotypes [27]. Those with pathogenic variants in desmosomal genes, intermediate filaments (e.g., desmin [DES], filamin C [FLNC]), and LMNA exhibit the highest incidence of sudden cardiac death and ventricular tachyarrhythmias (Table 2) [34]. Health professionals recommend those with desmosomal variant carriers to avoid or restrict their exercise, since vigorous exercise can worsen heart failure and cardiomyopathy [35].

The DCM Precision Medicine Study (DCM PM) was a family-based cross-sectional study that estimated the prevalence of familial DCM among probands and the risk among their first-degree relatives. It rigorously obtained phenotype and pedigree data on a large, multiracial idiopathic DCM cohort by identifying relevant genetic variants through exome sequencing and reporting results to probands and family members [36]. The central hypothesis of this study was that most DCM, whether familial or non-familial, has a genetic basis. Results demonstrated that first-degree relatives of non-Hispanic Black probands had increased risk of DCM, emphasizing the importance of clinical screening of at-risk family members in this population [37]. In addition, there was a strong correlation between patients’ genomic knowledge and trust in the medical researchers, underscoring the importance of patient education in genetics and genomics [38].

Precision medicine relies on managing disease risk among first-degree relatives of probands with a heritable disease; therefore, interventions to improve clinical screening are needed to improve outcomes. The DCM PM conducted a randomized controlled trial (RCT) to test the effectiveness of an educational booklet intervention, *Family Heart Talk*, for improving the uptake of preventive screening and surveillance in at-risk first-degree relatives [36,39]. Several sub-studies have been performed with the DCM PM cohort. Analysis of patients from DCM PM showed that an ischemic pattern of late gadolinium enhancement on MRI may be evident in patients with well-validated idiopathic DCM and does not necessarily reflect coronary artery disease [40]. Utility of testing for hereditary TTR variants also had low yield in patients with DCM without TTR-specific findings [41]. Further studies under the DCM PM cohort are being investigated.

### 6.2. Hypertrophic and Restrictive Cardiomyopathy

Genetic HCM is also defined by genetic and allelic heterogeneity [8]. Approximately 50% of HCM patients who go through genetic testing exhibit harmful variants in genes such as MYBPC3, MYH7, TNNT2, TNNI3, ACTN2, MYL3, and TPM1 [42]. These genes are associated with sarcomeric function and structure [42]. Individuals with these harmful sarcomere-positive genotypes have worse health outcomes such as earlier onset of HCM, higher risk for heart failure, atrial fibrillation, and ventricular arrhythmia than those with negative genetic testing or VUSs [43]. 

Genetic HCM has unpredictable expressivity and penetrance [8]. Only half of individuals who bear a hereditary sarcomeric variant developed HCM during a 15-year follow-up [42]. HCM penetrance may also differ between selected groups [8]. When analyzing data from participants with likely pathogenic or pathogenic sarcomeric variants, left ventricular hypertrophy penetrance was seen in only 18% [44]. This contrasts with the expected penetrance of a genotype-positive status that is correlated with harmful cardiovascular outcomes and increased wall thickness compared with those with the genotype-negative status [44]. Determining HCM penetrance between genotypes can be improved with further genetic screening and research [8].

Genetic testing in HCM is critical in characterizing HCM and sarcomeric disease and differentiating them from phenocopies including glycogen storage and lysosomal diseases and cardiac amyloidosis [8]. A study found that these phenocopies were identified in 1.45% of HCM patients who participated in genetic testing [19]. Genetic testing is necessary to detect these phenocopies, as medical management of the phenocopies is significantly different from that of sarcomeric HCM [8].

Cardiac amyloidosis is a common HCM mimicker [8]. Cardiac amyloidosis has become more recognized with the advent of genetic testing, especially with the TTR c.424G>A (p.Val142Ile) gene variant commonly found in patients with African ancestry [8]. Among patients initially diagnosed with HCM, 9% were found to have cardiac amyloidosis due to genetic testing [45]. Treatments for cardiac amyloidosis include transthyretin stabilizers for patients with TTR cardiac amyloidosis, and early clinical management has been linked with improved outcomes [46].

### 6.3. Left Ventricular Non-Compaction

Left ventricular noncompaction (LVNC) is characterized by a thinned, compact myocardial layer and a thickened trabecular myocardial layer with excessive trabeculations and deep recesses [47]. In the adult population, it has a prevalence of 50 per 100,000 [48]. LVNC can occur in isolation or may be associated with DCM, HCM, arrhythmia, congenital heart disease, and genetic syndromes including Duchenne Muscular Dystrophy and Barth Syndrome [49,50,51]. It has a wide spectrum in clinical presentation ranging from asymptomatic to severe cardiac dysfunction or sudden cardiac death [52]. Approximately 20 to 30% of LVNC patients have an underlying pathogenic variant identified with genetic testing [53]. Rojanasopondist et al. reviewed all genes previously reported to be associated with LVNC [45]. Among the 189 genes with prior reported association, only 32 genes were classified as having definitive or moderate evidence to support their relationship with LVNC after genetic pathway analysis. Many patients were also identified to have genetic variants related to other NICM, including DCM and HCM, adding to its complexity. Of the 32 genes identified in this study, one-third were related to sarcomere-related function, while the remainder included transcriptional or translational regulators, mitochondrial function, and cytoskeletal proteins. These wide variety of functions associated with these genes make it challenging to diagnose the underlying molecular pathway leading to LVNC [47]. Current evidence for genetic association with LVNC highlights the ambiguity and genetic heterogeneity of this disease, necessitating continued research to provide stronger evidence for familial screening and patient management.

## 7. Screening Strategies

### 7.1. Outreach Strategies

Strategies to increase cardiovascular genetic screening among first-degree relatives include sharing and educating probands with disease risk and progression [39]. Utilizing educational materials such as pamphlets and booklets has been found to increase genetic screening among family members [39]. These educational materials provide information on the risks of DCM and counseling for the proband’s family members [39]. Approximately 29.7% of probands have been found to have at least one first-degree relative with DCM [37]. Additionally, the overall risk of DCM in first-degree relatives was 19% by age 80, increasing to 33% when incorporating those with left ventricular enlargement or left ventricular systolic dysfunction [37]. These highlight the significance of screening first-degree relatives and educating probands and their family members on the disease and its clinical course.

Genetic counseling and education include sharing genetic risk information among family members [39]. This may be challenging due to a lack of health literacy, physical or emotional distance between relatives, and confidentiality issues [54]. Providers are not allowed to directly contact at-risk family members due to HIPAA and confidentiality regulations [54]. As a result, providers must emphasize the need for familial screening to probands, who should then encourage family members to be evaluated [39].

A study by Kinnamon and colleagues found that there is no statistically significant difference in relatives undergoing genetic screening when probands experience a face-to-face provider-led intervention versus paper or web-based formats [39]. Although the success outcomes are similar, provider-led interventions can be time-consuming [39]. This may be challenging in the absence of adequately trained genetic counselors in clinics [39]. Furthermore, results from the *Family Heart Talk* booklet mentioned above indicate that this intervention is efficient and cost-effective [39]. It is important to acknowledge that in this study, the DCM probands were enrolled in advanced heart failure programs, so the applicability of these results to DCM patients without advanced disease remains unknown [39]. The effectiveness of these educational strategies hinge on probands who are willing to enroll their relatives in genetic counseling [39].

### 7.2. Opportunistic Screening

Orthotopic heart transplantation remains the gold standard for treating patients with advanced heart failure [55]. Genetic testing for heart transplant recipients with prior cardiomyopathy has not been consistently performed [55]. It is a window of opportunity for genetic testing, and recent studies have shown its crucial role in identifying relatives at risk [55]. Approximately 60% of heart transplant recipients have an underlying etiology of non-ischemic cardiomyopathy [56]. Among patients with non-ischemic cardiomyopathies, heart transplant recipients are distributed as follows: DCM 50.8%, HCM 3.4%, and RCM 3.4% [56]. The clinical course of ARVC and LVNC eventually necessitates heart transplantation [56]. Retrospective genetic testing has not been regularly performed on heart transplant recipients with non-ischemic cardiomyopathy, but testing for genetic etiologies in these recipients can be crucial for relatives and family members [57].

Another study by Boen and colleagues evaluated the diagnostic yield of genetic testing in 31 heart transplant recipients with prior non-ischemic cardiomyopathy [57]. The study found a high diagnostic yield in the genetic testing results: 12 patients carried a class five variant (38.7%) and 11 patients carried a class three variant (35.5%) [57]. When accounting for genetic results pre-heart transplant, genetic diagnoses could be determined in 45.9% of all patients [57]. These results signify that genetic screening remains overlooked in patients with non-ischemic cardiomyopathies.

Early recognition of variant carriers and cardiac phenotypes ensures timely treatment, which leads to lower morbidity and mortality rates [57]. Among family members who are genotype-positive, approximately half manifest with abnormal cardiac evaluation even though they were asymptomatic [58]. Through genetic testing, these affected family members were identified early, leading to timely clinical care and treatment and preventing disease progression [58]. Segregation testing is another informative tool that can help reclassify variants for family members with VUSs [57]. Since the classification of VUSs can change, it is necessary for these patients to have longitudinal follow-ups and clinical checkups [57]. Changes in variant classification must be communicated to probands and their families [57].

Clinical genetic testing consideration is warranted beyond standard arrhythmogenic and cardiomyopathy gene variants when other causes leading to the need for advanced therapies may contribute [55]. For example, opportunistic genetic testing in patients with ischemic cardiomyopathy with early onset and/or strong family history can also be performed to identify familial hypercholesterolemia (FH) early on in heart transplant patients [55]. FH is the most common genetic disease linked to premature cardiovascular disease [55]. Early identification of FH can prevent severe disease progression [55]. It is concerning that FH remains genetically underdiagnosed, despite its adverse outcomes as early as the second decade of life [55]. If patients with FH are diagnosed early on, lipid-lowering drugs which significantly reduce their risk of atherosclerotic cardiovascular disease can be started sooner [55].

### 7.3. Risk Stratification 

Non-ischemic cardiomyopathy has a wide spectrum in clinical presentation, even within its sub-classifications of DCM, HCM, and RCM. Through the years, several risk stratification tools have been created and validated to help guide management of these patients. The HCM Risk-SCD calculator (www.hcmrisk.org (accessed on 18 May 2023)) was created by O’Mahony et al. in 2014 given the lack of international consensus on the absolute sudden cardiac death (SCD) risk that justifies ICD implantation. It has not been validated in pediatric patients, elite athletes, and individuals with metabolic diseases and clinical syndromes. This risk calculator includes age, left ventricular wall thickness, left atrial size, maximum left ventricular outflow track gradient, family history of sudden cardiac death, non-sustained ventricular tachycardia, and unexplained syncope [59]. An updated version is provided by the American Heart Association, which includes all the previously noted variables with the addition of left ventricular ejection fraction, apical aneurysm, and extensive late gadolinium enhancement on cardiac MRI. This calculator can be accessed at: https://professional.heart.org/en/guidelines-and-statements/hcm-risk-calculator (accessed on 18 May 2023).

Risk of life-threatening ventricular arrhythmias in patients with ARVC has been found to be predicted by four variables including younger age, male sex, burden of ventricular ectopy, and the extent of repolarization abnormalities. This risk model calculator (www.arvcrisk.com (accessed on 18 May 2023)) performed well in patients with and without pathogenic PKP2 variants and can guide clinicians in determining the timing of ICD implantation [60]. For patients with LVNC, a risk score model based on variables associated with major adverse cardiac events (MACE) was created by Casas et al. (www.lvnc-riskscore.com (accessed on 18 May 2023)). This prediction model has been externally validated to indicate that patients with normal ECG, preserved ejection fraction, no myocardial fibrosis, and no family aggregation do not have MACE during long-term follow-up [61]. Among LMNA patients, Wahbi et al. formulated a new score (https://lmna-risk-vta.fr/ (accessed on 18 May 2023)) to estimate the 5-year risk of life-threatening ventricular tachyarrhythmia in patients with LMNA mutation. A threshold of ≥7% predicted a 5-year 96% risk of long term ventricular arrhythmia among LMNA patients [61]. Phospholamban (PLN) p.Arg14del mutation carriers are at risk for malignant ventricular arrhythmia. An internally validated PLN 5-year risk calculator helps guide clinician assessment for primary prevention ICD placement by accounting for left ventricular ejection fraction, amount of inferior or precordial leads with negative T waves, low-voltage ECG, and amount of PVC/24 h [62]. These risk stratification models are constantly being updated as new data are available. 

## 8. Future Directions

Polygenic risk scores have been utilized to predict genetic risks in complex diseases such as coronary artery disease (CAD), schizophrenia, and cancer [63]. A PRS is the cumulative weighted sum of several common genetic variants throughout the entire genome [63]. This sum is established as a risk score and is used to identify individuals at high risk for certain diseases [63]. PRSs are being utilized to determine the penetrance of monogenic gene variants and can help interpret cardiac genetic tests for family members at risk [63]. Recent studies have shown how utilizing PRSs for CAD can stratify risk and improve clinical outcomes [63]. However, the evidence evaluating polygenic contribution to cardiomyopathy and HF development is very limited. One study did establish a PRS for HF in patients with CAD; this score was marked as HF-PRS [63]. The study found a statistically significant relationship between HF-PRS score and ischemic HF, allowing for risk stratification in CAD patients [63]. There is always a clinical need to better understand the genetic predisposition and basis for HF to detect and treat it early on. Future studies may allow for PRSs to evaluate genetic risk for HF and cardiomyopathy at any point in the patient’s lifetime and not just when associated with CAD. Since information on modifiable risk factors such as lifestyle and diet are continuously being collected, PRSs may determine absolute HF risk in the future [63]. With further engineering and technological advancements, PRSs have great potential to serve as a clinical risk stratification tool for HF and cardiomyopathy [63].

While technological advances have provided the opportunity for a whole-genome approach and different ways to identify risk markers and generate prediction algorithms, there are higher standards for such testing to become applicable in clinical practice. For example, the currently identified single nucleotide polymorphisms might not fully describe genetic diversity, as they may not capture known and unknown forms of genetic variability (e.g., copy number variation). Meanwhile, genetic mechanisms likely involve complex interactions among genes or between genes and their environmental exposures, or complex epigenetic or post-translational modification processes, that are not realized.

## 9. Conclusions

Genetic evaluation of cardiomyopathies plays an increasingly important role in the early diagnosis, treatment, and prognosis of disease. The main cardiomyopathies that genetic tests screen for are dilated, hypertrophic, arrhythmogenic, and restrictive cardiomyopathies, with left ventricular noncompaction representing a variant phenotype. It is important to recognize that human monogenic diseases may contribute to an unexpectedly larger portion of cardiomyopathies that we have previously recognized, as they may not be presented in a syndromic manner. The uncertainty surrounding the information of the discussed genetic tests should therefore be weighed against the risks of ignoring the potential of a malignant genetic predisposition that may adversely affect the patient and his/her affected family members, with the need for shared decision-making with the available clinical evidence. The early detection and establishment of therapies in patients with inherited cardiomyopathies optimize patient health and care. Not only does clinical genetic testing define the genetic basis of the cardiomyopathy and evaluate the prognosis, but it also allows for at-risk relatives to be screened and risk stratified. Clinical genetic evaluation allows for the guidance of risk stratification in relatives and should be managed by a multidisciplinary team to properly interpret cardiac genetic results, which will then alter the clinical care of patients and guide at-risk relatives. With this streamlined method, cascade testing can be performed to highlight at-risk family members and impactful genetic variants can be identified to establish personalized and specific treatment plans for patients. 

## Figures and Tables

**Table 1:** Variant Classification and Clinical Implications.

Variant Classification	Interpretation	Impact
Pathogenic (class V)	Positive, strong evidence that variant causes phenotype	Makes genetic basis of phenotype clearer. Can inform clinical care and management. Family members can be screened and risk-stratified through genetic testing data.
Likely pathogenic (class IV)	Positive, good evidence that variant causes phenotype, variant likely causes disease	
Variant of uncertain significance (VUS, class III)	Inconclusive result, more evidence is required to classify the variant as pathogenic or benign	Cannot clinically diagnose or manage due to uncertain result. Family testing not available. May be reclassified with more evidence.
Likely benign (class II)	Negative, variant is likely not associated with phenotype	Decreased chance that genetic variant is involved in disease, but does not fully dismiss the possibility of genetic etiology.
Benign (class I)	Negative, variant does not cause phenotype	

**Table 2:** Newly Recognized Non-Sarcomeric Genetic Causes of Dilated/Restricted Cardiomyopathies with Progressive Clinical Trajectories.

Gene	Gene-Specific Features
*RBM20*	RNA-binding motif protein 20 and splicing factor that targets cardiac genes (e.g., titin). Loss of RBM20 leads to proarrhythmic Ca^2+^ release from sarcoplasmic reticulum. Heterozygous missense mutation leads to clinically aggressive DCM with increased risk of malignant ventricular arrhythmias. Autosomal dominant inheritance. DCM penetrance is 66% and frequency is 1–5%.
*FLNC*	Codes for filamin C, a protein with structural and signaling functions in the myocyte, crosslinking actin filaments, and anchoring of sarcolemmal proteins to the cytoskeleton. May develop proximal skeletal muscle weakness and progressive DCM with increased risk of malignant ventricular arrhythmias. Autosomal dominant inheritance. Frequency is 1–4.5%.
*BAG3*	Codes for an anti-apoptotic co-chaperone protein located on sarcomere Z-disc. Regulates filamin production and clearance and myocyte contraction, protective mechanisms in DCM. Heterozygous mutations lead to disruption of myofibril structure/contractile function. Autosomal dominant inheritance. Frequency is 1–5%.
*PLN*	Codes for phospholamban, a protein inhibitor of cardiac SERCA2a in which phosphorylation relieves its inhibitory effects. Founder mutation (R14del) from the Netherlands associated with a high risk for malignant ventricular arrhythmias and progressive heart failure. Autosomal dominant inheritance. Frequency is 1–1.5%.
*DES*	Codes for desmin, a muscle-specific protein encoded by the DES genes that integrates sarcolemma, Z disc, and nuclear membrane in sarcomeres. Heterozygous mutations lead to DCM. Autosomal recessive inheritance. Frequency is 1–2%.

## Data Availability

No new data were created or analyzed in this study. Data sharing is not applicable to this article.
